# Assessment of energy loss in aortic stenosis using Bayesian multipoint phase-contrast MRI

**DOI:** 10.1186/1532-429X-15-S1-P47

**Published:** 2013-01-30

**Authors:** Christian Binter, Robert Manka, Simon H Sündermann, Verena Knobloch, Matthias Stuber, Sebastian Kozerke

**Affiliations:** 1 Institute for Biomedical Engineering, University and ETH Zurich, Zürich, Switzerland; 2 Dept. of Cardiology, University Hospital Zurich, Zurich, Switzerland; 3 Division of Cardiovascular Surgery, University Hospital Zurich, Zurich, Switzerland; 4 Center for Biomedical Imaging (CIBM), University of Lausanne, Lausanne, Switzerland; 5 Imaging Sciences and Biomedical Engineering, King's College London, London, UK

## Background

Aortic stenosis is the most prevalent valvular heart disease. The current gold standard for determination of the disease severity is Doppler echocardiography, but the decision for valve replacement is mostly based on the patient experiencing symptoms [1]. Doppler echocardiography measures the velocity across the valve, which is only indirectly linked to the energy loss associated with a diseased valve. It has recently been proposed that energy loss can be quantified directly by employing Bayesian multipoint phase-contrast MRI (PC-MRI) [2]. In this work we present preliminary data acquired in patients with severe aortic stenosis.

## Methods

A Bayesian multipoint velocity encoding sequence [3] was implemented on a 3T system (Philips Healthcare, Best, The Netherlands). A navigated and cardiac-triggered 3D gradient echo sequence with spatial and temporal resolution of 2.5 x 2.5 x 2.5 mm^3^ and 36 ms was used. A total of 10 velocity encoding points was acquired resulting in approximately 8 min scan time excluding navigator efficiency. Approval of the local Ethics Review Board was obtained and patients were recruited upon informed consent. Energy loss was computed based on the ratio of turbulent to mean kinetic energy taking reflow into account as proposed previously [2]. The pressure gradients were computed analogous to Doppler echocardiography using the modified Bernoulli equation: PG = 4v_max_^2^.

## Results

**Figure 1:**
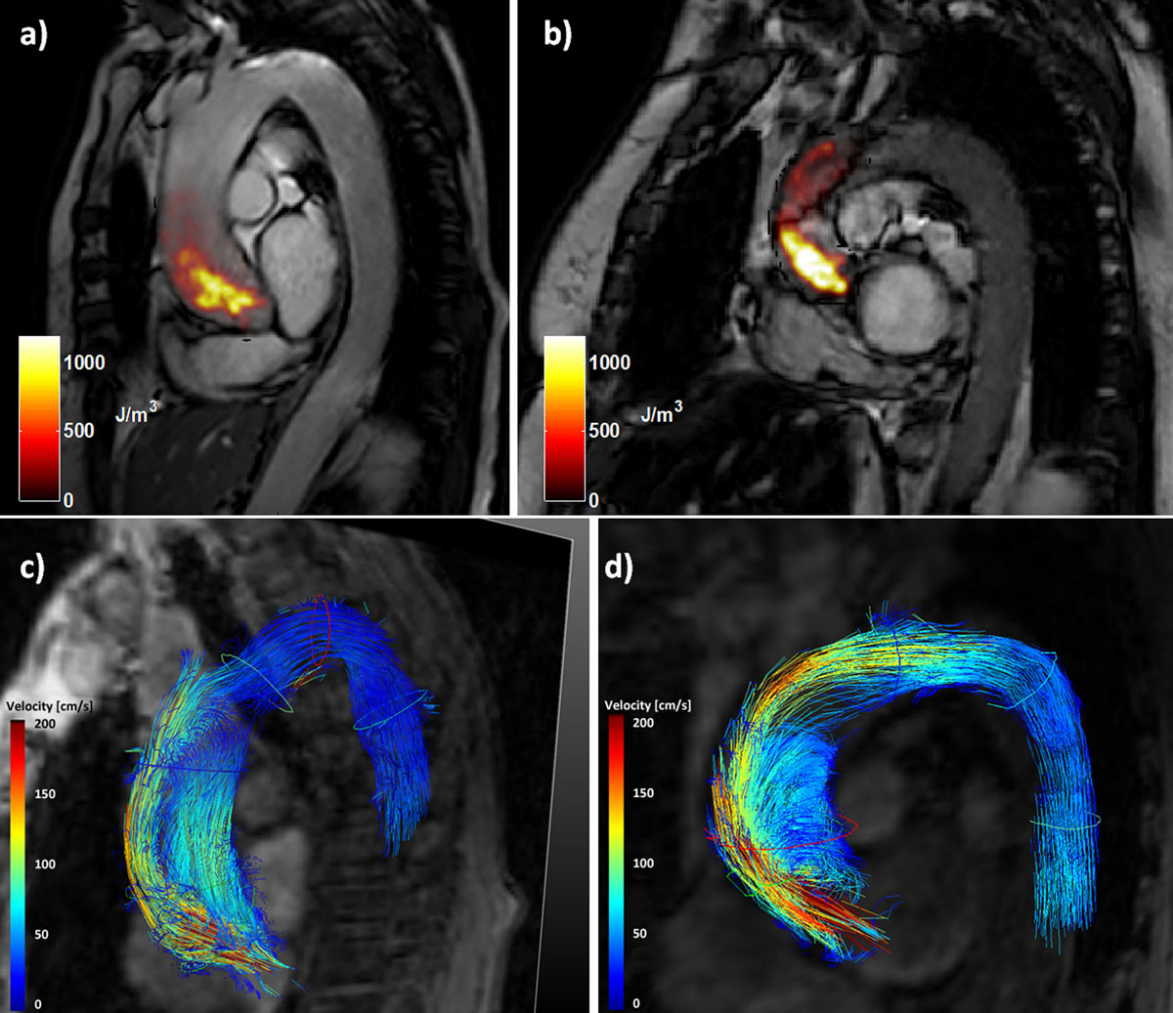
Maps of turbulent kinetic energy during systole in patient 1 (a) and patient 2 (b). Systolic flow pattern visualization using pathlines in patient 1 (c) and patient 2 (d). In both cases the majority of forward flow is directed along the outer wall of the aortic arch, and considerable recirculation in the ascending aorta occurs.

Preliminary data from two patients with symptomatic severe aortic stenosis referred for aortic valve replacement are presented. Peak velocities and turbulent kinetic energy (TKE) and energy loss indices (ELI) are listed in Table 1. Compared to our previous data obtained in healthy subjects [3], peak TKE in the patients presented here was found to be significantly increased (149±12 J/m^3^ vs. 1350 and 1630 J/m^3^, respectively). It is noteworthy that the patient with higher TKE values had a lower energy loss index. Values from Doppler echocardiography are given for comparison. Figure 1 shows maps of TKE in the aortic arch in both patients along with flow patterns derived from the velocity data.

**Table 1:** Flow parameters for both patients as determined by MRI and Doppler echocardiography.

	Patient 1	Patient 2
***Bayesian multipoint PC-MRI***		

Peak velocities	3.9 m/s	4.5 m/s
Peak TKE	1350 J/m^3^	1630 J/m^3^
Peak total TKE	12.1 mJ	15.3 mJ
Energy loss index	22.9%	21.9%
Stroke volume	56 ml	63 ml
Mean pressure gradient	45 mmHg	41 mmHg

***Doppler echocardiography***		

Aortic valve area	0.8 cm^2^	0.8 cm^2^
Mean pressure gradient	48 mmHg	42 mmHg

## Conclusions

Bayesian multipoint PC-MRI permits concurrent mapping of both mean kinetic and turbulent kinetic energy in patients and allows the assessment of relative energy loss and pressure gradients associated with aortic valve stenosis. The energy loss index was found to be approximately 8-fold higher as compared to healthy subjects [2] and may hold promise to serve as a novel marker for grading valve disease.

## Funding

Christian Binter was supported by the National Competence Center in Biomedical Imaging Switzerland.
